# Prospective Analysis of Lipid Composition Changes with Antiretroviral Therapy and Immune Activation in Persons Living with HIV

**DOI:** 10.20411/pai.v2i3.218

**Published:** 2017-10-06

**Authors:** Martha A. Belury, Emily Bowman, Janelle Gabriel, Brandon Snyder, Manjusha Kulkarni, Marilly Palettas, Xiaokui Mo, Jordan E. Lake, David Zidar, Scott F. Sieg, Benigno Rodriguez, Martin P. Playford, Adriana Andrade, Daniel R. Kuritzkes, Nehal N. Mehta, Michael M. Lederman, Nicholas T. Funderburg, the AIDS Clinical Trials Group A5248/A5249s Team

**Affiliations:** 1 Department of Human Sciences, Ohio State University, Columbus, Ohio; 2 School of Health and Rehabilitation Sciences, Division of Medical Laboratory Science, Ohio State University, Columbus, Ohio; 3 Center for Biostatistics, Department of Biomedical Informatics, Ohio State University, Columbus, Ohio; 4 University of Texas Health Science Center, Houston, Texas; 5 Case Western Reserve University, Cleveland Ohio; 6 National Heart Lung and Blood Institute, Bethesda, Maryland; 7 Johns Hopkins University, Baltimore, Maryland; 8 Brigham and Women's Hospital/Harvard Medical School, Boston, Massachusetts

**Keywords:** lysophosphatidylcholine, immune activation, antiretroviral therapy, fatty acids

## Abstract

**Background:**

Lipid profiles are altered by HIV infection and antiretroviral therapy (ART). Among HIV-uninfected (HIV-) populations the concentrations of various lipid classes (ie, lysophosphatidylcholine, LPC) and their saturated (SaFA), monounsaturated (MUFA), and polyun-saturated fatty acid (PUFA) composition are related to cardiometabolic disease risk. Associations between changes in the lipidome and immune activation in HIV-infected (HIV+) individuals beginning ART have not been described.

**Methods:**

Plasma lipid concentrations and their fatty acid composition were measured by differential mobility spectroscopy in samples from 35 treatment-naive HIV+ participants beginning raltegravir (RAL)-based ART and from HIV- individuals (n = 13) matched for age and sex.

**Results:**

The levels of SaFA, including palmitic (16:0) and stearic (18:0) acid were enriched in HIV+ participants (pre- and post-ART), and SaFA levels were often positively correlated with levels of immune activation (ie, IL-6, sCD14, and TNFR1) at baseline and week 48. Levels of PUFAs (including 18:3, 20:4, and 20:5) were lower in HIV+ participants at baseline compared to levels in HIV- participants (*P* < 0.01), and levels of these PUFAs were increased following 48 weeks of ART. Levels of PUFAs were often inversely related to immune activation. Levels of LPC were increased in HIV+ participants, both pre- and post-ART vs HIV- participants, and the composition of LPC was enriched for SaFAs among HIV+ individuals. At week 48, several LPC molecules containing SaFAs were positively correlated with levels of sCD14, D-dimer, and TNFR1 (*P* < 0.01), and levels of PUFA-containing LPC (18:3, 20:5, 22:5, 22:6) were positively correlated with CD4+ T cell counts and inversely correlated with sCD14 and IL-6 (*P* < 0.01).

**Conclusions:**

The composition of the lipidome is altered in HIV infection and changes when ART is administered. Alterations in SaFAs were generally associated with inflammatory markers and may contribute to comorbid disease pathogenesis.

## INTRODUCTION

Human immunodeficiency virus (HIV) infection, its treatment, and the inflammatory consequences of chronic HIV infection all contribute to cardiometabolic disease risk [1, 2]. Chronic HIV infection is often associated with decreases in levels of high-density lipoprotein (HDL) cholesterol and increases in low-density lipoprotein (LDL) cholesterol, triglycerides (TG), and total cholesterol (TC) [1, 2]; however, these basic lipid panels provide only a crude characterization of blood lipids and are insufficient to accurately assess cardiovascular disease (CVD) risk in individuals infected with HIV [3]. Historically, assays of lipid families have aggregated hundreds of different molecular species into classes based on their densities; newer methods now allow finer characterization of these elements.

Fatty acid concentrations and composition in plasma, including levels of saturated (SaFA), monounsaturated (MUFA), and polyunsaturated fatty acids (PUFA) have been associated with CVD [4, 5], non-alcoholic steatohepatitis (NASH) [6] and diabetes [7] in HIV- populations. For example, SaFAs, especially palmitic acid, are associated with increased risk of type 2 diabetes in an HIV- population [7]. Increased levels of PUFAs are associated with reduced risk of myocardial infarction [8] and death [9]. Furthermore, *in vitro* studies have demonstrated that SaFAs, but not unsaturated fatty acids, can activate the inflammasome within myeloid cells [10], can induce NFKB signaling in macrophages [11], and can promote adhesion molecule expression on endothelial cell lines [12]. Conversely, PUFAs can block activation of the inflammasome [13, 14] and inhibit endothelial cell activation following exposure to lipopolysaccharide (LPS) [15].

Lysophosphatidylcholine (LPC) levels are increased in many disease settings, including CVD [16, 17], renal failure [18], and diabetes [19]. Based on its fatty acid composition, LPC can act as a pro- or anti-inflammatory molecule [17]. The relationship between inflammation and lipids is bidirectional [20], with inflammatory processes altering lipid levels, and several lipid species contributing to inflammation and cellular activation. Lysophosphatidylcholine is a major component of oxidized LDL (oxLDL) [21], which can activate monocytes *in vitro* [22, 23], and we have previously reported that oxLDL levels are increased in HIV-infected (HIV+) individuals and are associated with markers of monocyte activation [24]. Reductions in oxLDL levels are associated with improvements in carotid intima media thickness [25] and non-calcified plaque volume [26] in HIV+ individuals receiving statins.

Here, in a substudy of the AIDS Clinical Trials Group (ACTG) trials A5248/A5249s [27–30], using liquid chromatography tandem mass spectrometry (LC MS/ MS; Complex Lipid Panel, Metabolon), we examined the concentrations and fatty acid composition of lipid classes in plasma samples: 1) longitudinally in ART-naive HIV+ adults who initiated an ART regimen containing raltegravir (RAL), tenofovir (TDF), and emtricitabine (FTC), and 2) in samples from a single time point in an age and sex similar HIV- population. We hypothesized that infection with HIV and treatment with ART would alter the composition of the lipidome, and that these changes would be associated with markers of inflammation.

## METHODS

### Ethics Statement

This study was approved by institutional review boards at all participating sites [27–30] and is registered with Clinicaltrials.gov (NCT00660972).

### Study Design

This pilot study A5248 was a prospective, open-label, multicenter study performed in the United States of America between June 2008 and April 2010 [27, 28]. Briefly, ART-naive subjects (n = 39) with plasma HIV-1 RNA viral load (VL) > 10k and < 300k copies/mL at screening were treated for 48 weeks with RAL (400 mg twice daily) and FTC/TDF (200 mg / 300 mg once daily). We selected 35 participants for this substudy who had available plasma samples at baseline (BL) and week 48 for analysis of their lipidome; all selected participants achieved virologic suppression by week 48. Samples from 13 age- and sex-matched HIV- subjects were analyzed for comparison.

### Sample collection

Blood samples were collected in EDTA-containing tubes, centrifuged once, and plasma was frozen at -80°C until thawed and analyzed in batches.

### Measurement of the lipid metabolites

Measurement of the concentration and composition of approximately 1,300 lipid metabolites was performed by Metabolon (True Mass Complex Lipid Panel, Metabolon, Research Triangle Park, North Carolina). Lipids were extracted from plasma in methanol:dichloromethane in the presence of internal standards. The extracts were concentrated under nitrogen and reconstituted in 0.25 mL of 10mM ammonium acetate dichloromethane:methanol (50:50). The extracts were transferred to inserts and placed in vials for infusion-Mass Spectroscopy (MS) analysis, performed on a Shimazdu LC with nanoPEEk tubing and the Sciex SelexIon-5500 QTRAP. The samples were analyzed via both positive and negative mode electrospray. Lipid class concentrations (μM) were calculated from the sum of all molecular species within a class, and fatty acid compositions (mol%) were determined by calculating the proportion of each class comprised by individual fatty acids. The Surveyor program was used in data organization and provides the name for the most common/abundant fatty acid measured.

The Mehta laboratory performed advanced lipid phenotyping using NMR spectroscopy (Liposcience, Raleigh, NC) for lipid concentration, lipid particle size, and number as reported previously [29]; the lipoprotein insulin resistance (LIPR) score [31] was calculated by Liposcience Labcorp, Burlington, NC. The LPIR score combines information from large VLDL-P, small LDL-P, large HDL-P, VLDL, HDL and LDL particle sizes into a weighted algorithm that can be used to measure insulin resistance. A score of 0 is insulin sensitive, 100 is insulin resistant, and the 50th percentile has an LPIR of 45.

### Flow cytometry

CD4+ and CD8+ T cells were identified by size, granularity, and staining with antibodies to CD4 or CD8. The following antibody-fluorochrome conjugates (and isotype controls) were used: anti-CD4 (Pacific Blue, Becton Dickinson (BD) Pharmingen, San Diego, CA), anti-CD8 (Peridinin-chlorophyll-protein Complex, PerCP, Franklin Lakes, NJ), anti-Ki-67 (phycoerythrin, PE, BD Pharmingen), anti HLA-DR (fluorescein isothiocyanate, FITC, BD Biosciences), and anti-CD38 (PE, BD Biosciences). For analysis of intracellular Ki-67, cells were incubated with FACS Permeabilizing Solution (BD Biosciences) for 15 minutes, washed and then stained with anti-Ki-67 antibody or with an isotype control antibody for 45 minutes in the dark. Cells were then washed and fixed with 1% formaldehyde and analyzed using an LSR II flow cytometer (BD).

### Plasma biomarker assays

Levels of soluble CD14 (sCD14), tumor necrosis factor receptor type 1 (TNFR1), and interleukin-6 (IL-6) were measured using Quantikine ELISA kits (all from R&D Systems Minneapolis, MN). Levels of D-dimer were measured using the Asserachrom D-DI immunoassay (Diagnostica Stago, Asnieres, France). Levels of leptin and adiponectin (R&D Systems) were also measured by ELISA.

### Statistical Methods

Fisher's Exact tests were used to test the association between HIV status and race or sex. The differences in the concentration and composition of total, free, and LPC lipid levels and age differences among HIV+ and HIV- donors were analyzed by 2-sample *t* tests, and the differences in these metabolic indices between baseline and week 48 of ART were analyzed by paired *t* tests. The associations among the levels of lipids and markers of immune activation were assessed by using Pearson correlations. Data analysis was performed in SAS 9.4 (SAS, Inc; Cary, NC).

## RESULTS

We included in this study longitudinal samples from 35 HIV+ participants (untreated and after 48 weeks of ART) and, for comparison, samples from thirteen HIV- participants at a single time point. Demographic information is shown in Table 1. Among the HIV+ participants at baseline, the median CD4+ T-cell count and viral load were 259 cells/μL and 37,153 copies/mL; both of these indices improved after week 48 of ART (to 489 cells/μL and < 48 copies/mL). Levels of LDL, HDL, TC, and TG are also shown in Table 1. As we have reported previously [29], among the HIV+ participants, median levels of LDL, HDL, TC, and TG all increased from baseline to week 48 (Table 1). Levels of adiponectin and leptin did not change significantly following 48 weeks of ART. The LPIR scores were slightly higher in HIV+ participants at baseline (*P* = 0.06) but were not different following 48 weeks of ART (*P* = 0.2) compared to LPIR scores among HIV- participants. Of the 32 HIV+ participants of whom we measured an LIPR score, 12 had an increase in their score following 48 weeks of ART; increases in this score have been reported to predict development of type 2 diabetes mellitus [32].

**Table 1.** Demographics and clinical characteristics of HIV-uninfected individuals and participants enrolled in A5248, at baseline and following 48 weeks of ART. Data displayed are medians and ranges, unless otherwise specified.

	HIV-1 infected participants Baseline (n = 35)	HIV-1 infected participants Week 48 (n = 35)	HIV-1 uninfected participants (n = 13)	Statistically different betweengroups
Age (years)	43 (23-58)	44 (24-59)	43 (23-58)	*P* = 0.98
CD4+ T-cell count (cells/μL)	259 (1-599)	489 (87-1026)	NA	
HIV Viral load (copies/mL)	37,153 (6,637-619,797)	48 (48-48)	NA	
Race/Ethnicity (%)	White = 18 (53%) Not White = 16 (47%)		White = 9 (69%) Not white = 4 (31%)	*P* = 0.35
Sex/Gender(%)	Male = 32 (91%) Female = 3 (9%)		Male = 10 (77%) Female = 3 (23%)	*P* = 0.32
Total cholesterol (mg/dL)	125 (87-208)	142 (93-226)	185 (93-266)	*P* < 0.001, HIV-vs BL *P*=0.005 HIV-vs Wk48 *P*= 0.004, BL vs Wk48
LDL (mg/dL)	73 (51-154)	84 (45-164)	100 (34-176)	*P* = 0.02, HIV-vs BL *P*=0.17, HIV-vs Wk48 *P*= 0.02, BL vs Wk48
HDL (mg/dL)	35 (0-56)	41 (17-71)	57 (41-103)	*P* < 0.001, HIV-vs BL *P*< 0.001, HIV-vs Wk48 *P*= 0.004, BL vs Wk48
Triglycerides (mg/dL)	72 (36-162)	87 (30-200)	89 (62-205)	*P* = 0.48, HIV- vs BL P=0.95, HIV- vs Wk48 *P*= 0.34, BL vs Wk48
Adiponectin (ng/mL)	4,281 (558-14,652)	3,599 (445-8,862)	NA	*P* = 0.1
Leptin (pg/mL)	2,412 (324-81,415)	5,855 (76-79,114)	NA	*P* = 0.06
Lipoprotein insulin resistance (LPIR) score	52 (10-77)	53 (10-86)	42 (1-70)	*P* = 0.06, HIV- vs BL *P*=0.2, HIV- vs Wk48 *P*= 0.42, BL vs Wk48

**The concentrations of total and free saturated and unsaturated fatty acids are altered in HIV infection and by ART.** The concentrations of several fatty acids differed significantly between treatment-naive HIV+ (baseline) and HIV- participants (Supplemental Table 1A, 1B). The overall concentration of free (non-esterified) fatty acids was decreased in HIV+ participants (298.8 μM) at baseline compared to levels in HIV- participants (442.6 μM, *P* = 0.03); these levels increased by week 48 of ART and were no longer significantly different (351.9 μM, *P* = 0.12). Levels of free oleic acid (18:1) and levels of several free PUFAs (linoleic acid 18:2, α-linoleic acid 18:3, docosatetraenoic acid 22:4, DPA 22:5, and DHA 22:6) tended to be lower in HIV+ baseline samples. Total fatty acid concentrations of arachidonic acid (20:4) and EPA (20:5) were also lower in HIV+ participants at baseline. Following 48 weeks of ART, levels of many free and total SaFAs, MUFAs, and PUFAs changed from baseline. Levels of free PUFAs (docosatetraenoic acid, DPA, and DHA) and levels of total arachidonic acid and EPA increased significantly from baseline and were no longer different from levels in HIV- participants (Supplemental Table 1A, 1B).

**Figure 1.**
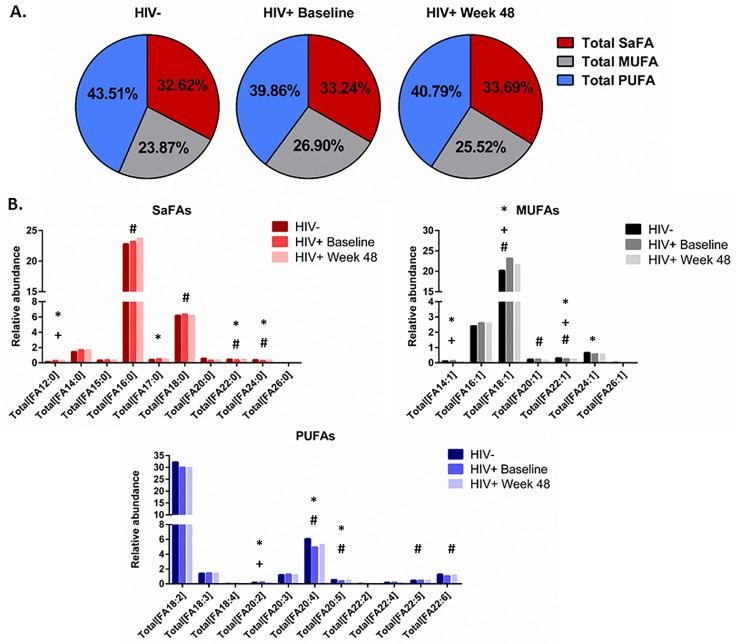
**The fatty acid composition of total fatty acids is altered in HIV infection at baseline and week 48 compared to levels in HIV-uninfected participants.** Plasma samples were thawed and the proportional representation (mol%) of A) SaFAs, MUFAs, and PUFAs and B) individual lipid molecules among total lipids, were measured by the Complex Lipid Panel (Metabolon). Several changes in the proportional representation of SaFAs, MUFAs, and PUFAs were measured among samples from HIV- participants, and in samples from HIV+participants pre-ART initiation (baseline) and following 48 weeks of ART. Mean levels are reported. Statistically significant differences among the subject groups are designated; an exploratory value of *P* < 0.05 is used for statistical significance (or something similar).

**Figure 2.**
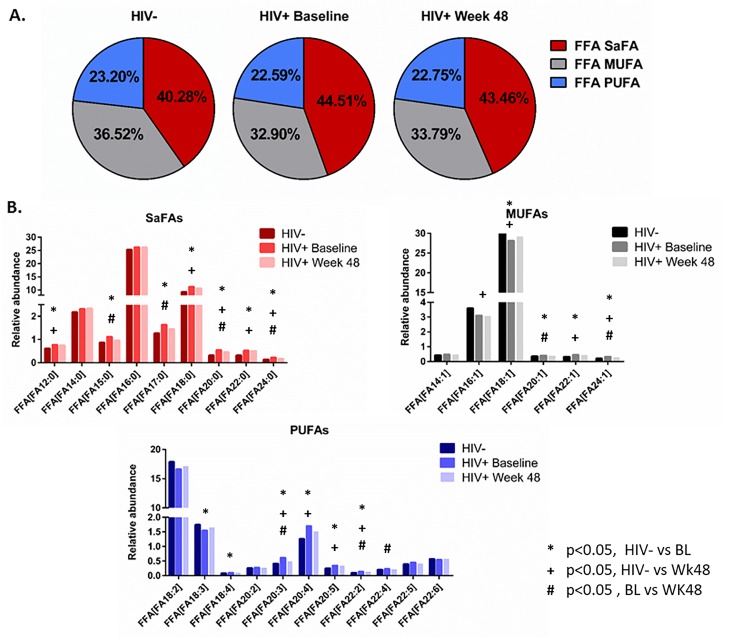
**The fatty acid composition of free fatty acids is altered in HIV infection at baseline and week 48 compared to levels in HIV-uninfected participants.** Plasma samples were thawed and the proportional representation (mol%) of A) SaFAs, MUFAs, and PUFAs and B) individual lipid molecules among “free” (non-esterified) lipids, were measured by the Complex Lipid Panel (Metabolon). Several changes in the proportional representation of SaFAs, MUFAs, and PUFAs were measured among samples from HIV- participants, and in samples from HIV+ participants pre-ART initiation (baseline) and following 48 weeks of ART. Mean levels are reported. Statistically significant differences among the subject groups are designated; an exploratory value of *P* < 0.05 is used for a cutoff of significance.

**The saturated and unsaturated fatty acid compositions of total and free fatty acids are altered in HIV infection and by ART.** We also report that the composition of total and free fatty acids in plasma differed in HIV- and treatment-naive HIV+ participants. Several SaFAs were enriched among total and free fatty acids in HIV infection at baseline compared to levels in HIV- participants (Figures 1 and 2, and Supplemental Tables 2A, 2B), including stearic acid. By comparison, several MUFAs and PUFAs were lower in HIV+ participants at baseline compared to levels in HIV- participants (Figures 1 and 2, and Supplemental Tables 2A, 2B) including free oleic acid (18:1) and α-linoleic acid (18:3) and total EPA (20:5). Many of these fatty acids (eg, stearic, arachidic, and behenic acid) decreased following ART; however, these values remained increased compared to levels measured in HIV- participants. Furthermore, levels of several total PUFAs (eg, EPA 20:5, DPA 22:5, and DHA 22:6) increased significantly from baseline following 48 weeks of ART (Figure 1B and 2B and Supplemental Table 2A, 2B).

**Concentrations of total and free fatty acids are associated with markers of immune activation in HIV infection.** We observed several relationships among the concentrations of free and total fatty acids and indices of immune activation in HIV+ participants. At baseline, concentrations of many free fatty acids: myristic acid (14:0, r = 0.35, *P* = 0.04), stearic acid (18:0, r = 0.36, *P* = 0.04), docosatetraenoic acid (22:4, r = 0.41, *P* = 0.01), and DPA (22:5, r = 0.48, *P* = 0.004) were directly related to CD4+ T- cell counts. Among total fatty acids, the concentrations of palmitic acid (16:0, r = 0.49, *P* = 0.003) stearic acid (18:0, r = 0.47, *P* = 0.005), linoleic acid (18:2, r = 0.52, *P* = 0.001), α-linoleic acid (18:3, r = 0.53, *P* = 0.001), and EPA (20:5, r = 0.35, *P* = 0.04) were positively correlated with sCD14 levels. The concentrations of total myristic (14:0) and pentadecylic acid (15:0) were associated with both IL-6 and TNFR1 levels. The concentration of total palmitic acid (16:0) was also related to levels of IL-6 (r = 0.33, *P* = 0.05) and TNFR1 (r = 0.39, *P* = 0.002), and the concentration of stearic acid (18:0) was also related to levels of TNFR1 (r = 0.38, *P* = 0.02). The total fatty acid concentrations of vaccenic acid (18:1) and α-linoleic acid (18:3) were directly related to levels of TNFR1; total concentrations of 20:1, 20:2, 20:3, and 22:5 were also directly related to TNFR1 (*P* < 0.05 for all).

**Figure 3.**
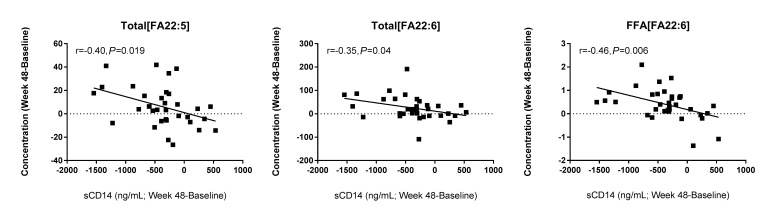
**Changes in PUFA levels are associated with changes in sCD14 levels following ART.** Plasma samples were thawed and levels of sCD14 were measured by ELISA. Greater increases in PUFA levels were associated with greater decreases in sCD14 levels. Pearson correlations among the changes in these indices (Week 48 - baseline) are reported.

Following 48 weeks of ART, several other relationships were identified. Among total fatty acids, the concentration of linoleic acid (18:2, r = -0.42, *P* = 0.015), α-linoleic acid (18:3, r = -0.31, *P* = 0.08), EPA (20:5, r = -0.31, *P* = 0.07), and erucic acid (22:1, r = -0.45, *P* = 0.007) tended to be inversely associated with levels of IL-6. Similarly, the free fatty acid concentration of EPA was also inversely related to IL-6 (r = -0.37, *P* = 0.04). Furthermore, following 48 weeks of ART, greater increases in the concentrations of total DPA (r = -0.40, *P* = 0.019), total DHA (r = -0.35, *P* = 0.04), and non-esterified DHA (r = 0.46, *P* = 0.006) were associated with greater declines in sCD14 (Figure 3).

**The fatty acid composition of total and free fatty acids are associated with markers of immune activation in HIV infection.** At baseline, the fatty acid composition of both free and total fatty acids were related to markers of immune activation and disease progression. The proportional representation of arachidonic acid (20:4) among total fatty acids was directly related to CD4+ T-cell counts (r = 0.35, *P* = 0.04) and inversely with viremia (-0.38, *P* = 0.025) and TNFR1 levels (-0.36, *P* = 0.03). The proportional representations of linoleic acid (18:2), erucic acid (18:1), and arachidonic acid (20:4) were all inversely related to levels of IL-6 and TNFR1 (*P* < 0.05).

Following 48 weeks of ART, the proportional representation of palmitic acid (16:0) among the total fatty acids tended to be positively associated with levels of IL-6 (r = 0.43, *P* = 0.01), TNFR1 (r = 0.32, *P* = 0.06), and sCD14 (r = 0.32, *P* = 0.07). Among free fatty acids, the proportional representation of palmitic acid (16:0) was related to levels of TNFR1 (r = 0.41, *P* = 0.01). The proportional representations of free lauric acid (12:0) and erucic acid (22:1) were also directly related to levels of sCD14 at week 48 (*P* < 0.01).

**The concentration and the fatty acid composition of LPC is altered in HIV infection and following ART.** We next focused on changes in the overall concentration and composition of LPC, as increased levels of this lipid class are associated with CVD [16, 17], renal failure [18], and diabetes [19]. At both baseline and after 48 weeks of ART, the concentration of LPC was greater in HIV+ participants than levels among uninfected controls (202, 213.7, and 184 μM, respectively *P* = 0.003 and 0.002). The concentration of LPC did not change significantly after 48 weeks of ART.

**Figure 4.**
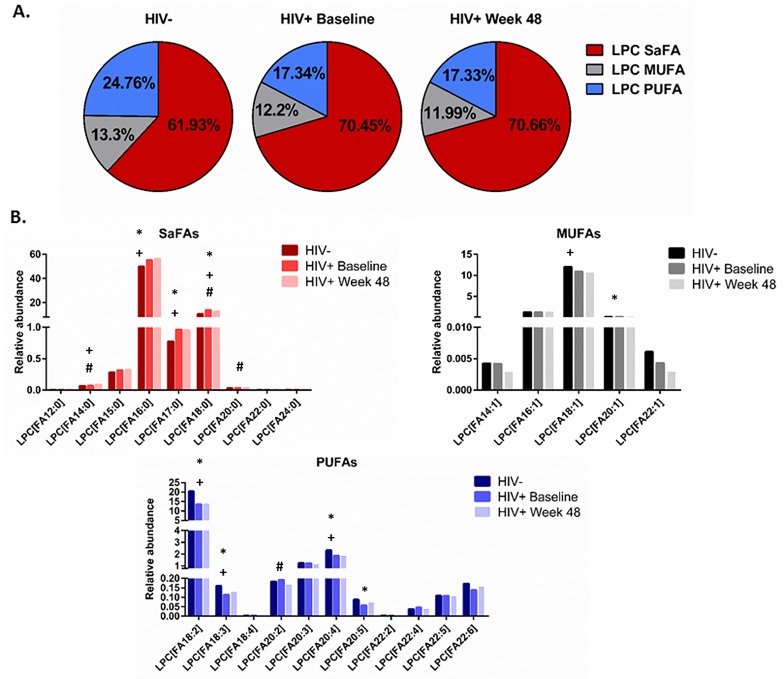

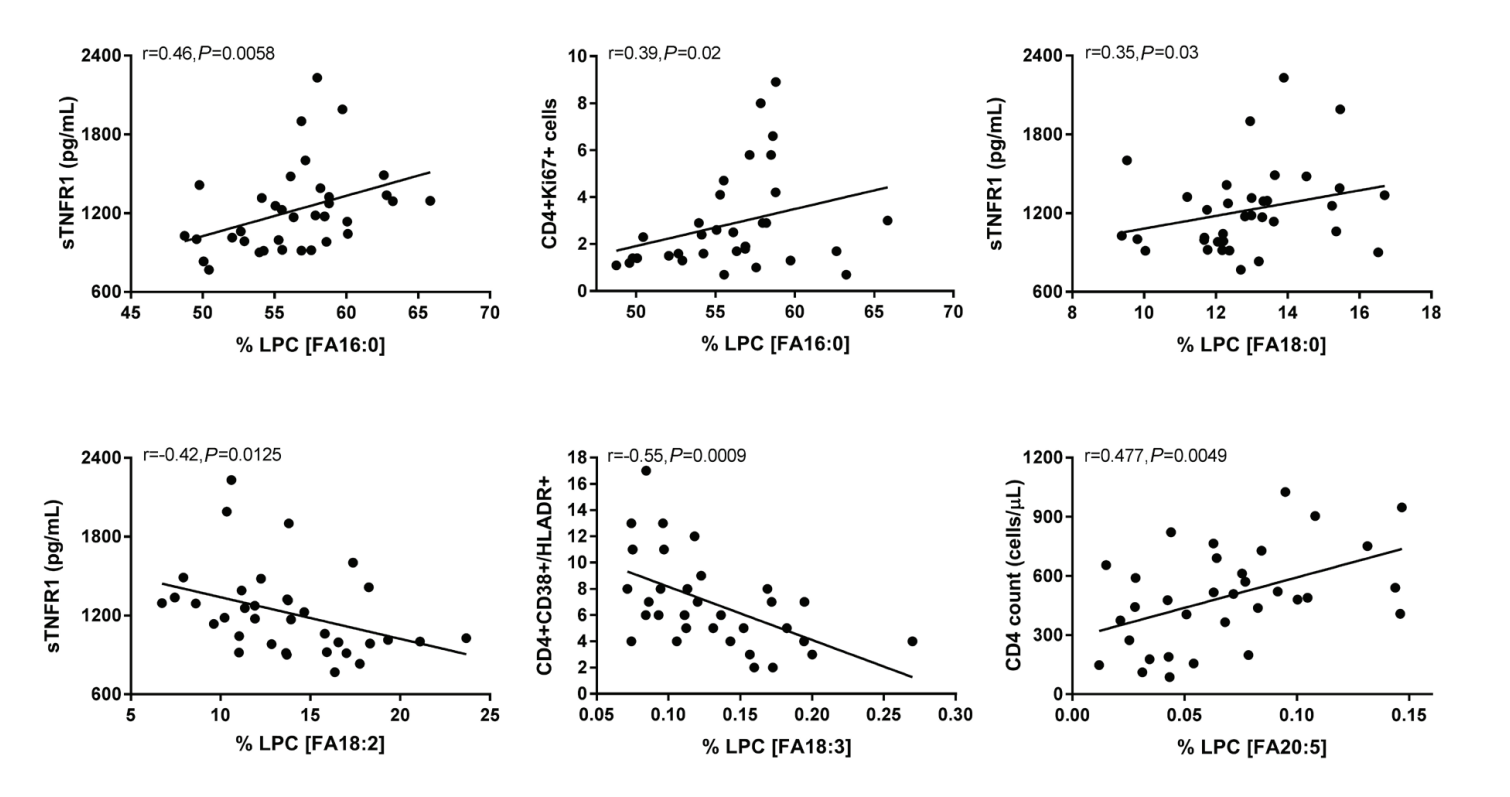

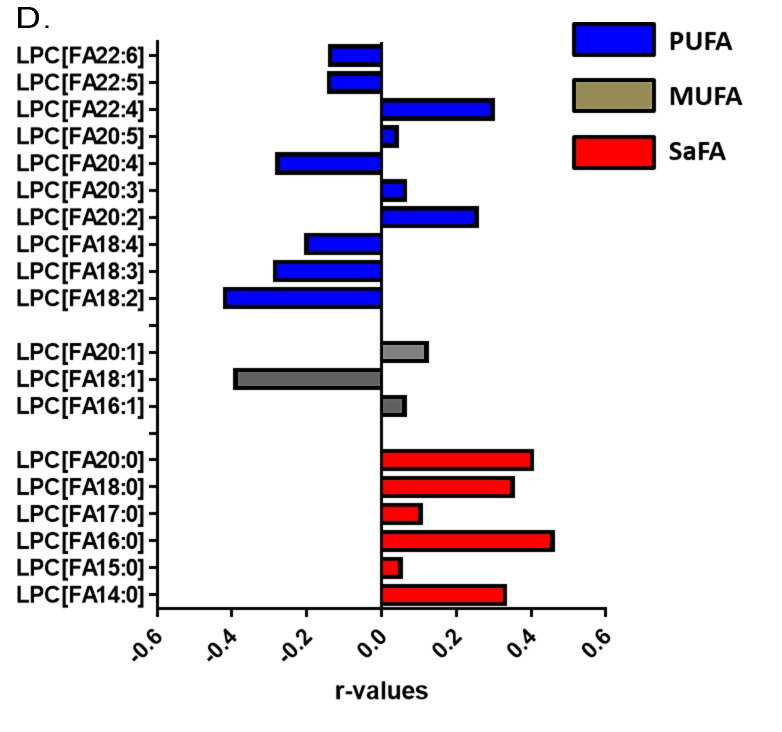
**The fatty acid composition of LPC is altered in HIV infection at baseline and week 48 compared to levels in HIV-uninfected participants and is associated with markers of immune activation.** Plasma samples were thawed and the proportional representation (mol%) of A) SaFAs, MUFAs, and PUFAs and B) individual lipid molecules among the lysophosphatidylcholine lipid class were measured by the Complex Lipid Panel (Metabolon). Several changes in the proportional representation of SaFAs, MUFAs, and PUFAs were measured among samples from HIV- participants, and in samples from HIV+ participants pre-ART initiation (baseline) and following 48 weeks of ART. Mean levels are reported. C) Plasma and cryopreserved peripheral blood mononuclear cell (PBMC) samples were thawed and levels of immune activation were measured by ELISA or by flow cytometry. Pearson correlation values are reported for relationships among immune activation/disease progression markers and lipid levels. Statistically significant differences among the participant groups are designated; an exploratory value of *P* < 0.05 is used for a cutoff of significance. D) The fatty acid composition of LPC molecules is associated with levels of immune activation, including TNFR1. At 48 weeks, LPC molecules containing SaFAs (red) are directly related to levels of TNFR1; levels of PUFA containing LPC molecules (blue) are typically inversely related to TNFR1 levels.

The fatty acid composition of LPC is important for its role in driving inflammation [17, 33, 34]. Therefore, we next asked if the fatty acid composition of LPC was altered by HIV infection and/ or with ART. Overall, in HIV + participants at baseline and week 48, the fatty acid composition of LPC was enriched for SaFAs (70.45% and 70.66%, respectively) compared to that of HIV- participants (61.93%, *P* < 0.001 for both); we also measured a significantly lower proportion of PUFAs in LPC among HIV+ participants at baseline and week 48 (17.34% and 17.33%) compared to the proportion of PUFAs within LPCs in HIV- participants (24.76%, *P* < 0.001 for both, Figure 4A). Furthermore, the composition of LPC was enriched for specific SaFAs: lauric acid (12:0), palmitic acid (16:0), stearic acid (18:0), and arachidic acid (20:0) in HIV+ participants at baseline and at week 48 compared to the composition of LPC in HIV- participants (Figure 4B and Supplemental Table 3B). Conversely, the composition of LPC in HIV- participants was enriched for MUFAs and PUFAs, including: octadecaenoic acid (18:1), eicosenoic acid (20:1), linoleic acid (18:2), arachidonic acid (20:4), and EPA (20:5), compared to the composition of LPC in HIV+ participants at baseline and following 48 weeks of ART (Figure 4B). Treatment for 48 weeks with ART resulted in significant increases in the proportions of LPC molecules containing the SaFAs palmitic acid (16:0) and arachidic acid (20:0) and the PUFA eicosadienoic acid (20:2) compared to baseline. Treatment with ART reduced the proportional representation of stearic acid (18:0) among LPC molecules, but these levels remained increased compared to levels in HIV- participants. The concentrations of several SaFA-containing LPC molecules were increased in HIV+ participants at both baseline and following 48 weeks of ART, compared to levels in uninfected participants (Supplemental Table 3A).

**Relationships exist among the concentration and composition of saturated and unsaturated fatty acid containing LPC and markers of inflammation.** At baseline, the plasma concentration (μM) of LPC 20:5 tended to be inversely associated with viremia (r = -0.3, *P* = 0.07). Also at baseline, the proportional representation of LPC 16:1 was directly related to IL-6 levels (r = 0.44, *P* = 0.009) and the proportion of LPC 22:4 tended to be inversely related to the proportion of CD4+ T cells co-expressing CD38 and HLA-DR (r = -0.32 *P* = 0.07).

Following 48 weeks of ART, we found more relationships among plasma concentrations of LPC molecules, the proportional representation of fatty acids within LPC, and markers of immune activation. The plasma concentrations of LPC 14:0 (r = 0.4, *P* = 0.02) and LPC 20:0 (r = 0.37, *P* = 0.03) were directly related to sCD14 levels; and the concentration of several PUFA containing LPCs (18:2, 18:3, 20:4, 20:5, 22:5, 22:6) were inversely related to levels of IL-6 (*P* < 0.05 for all). Plasma concentrations of LPC 20:4, 20:5, 22:5, and 22:6, were directly related to CD4+ T-cell counts (*P* < 0.05, for all). The proportional representation (composition, mol%) of several SaFA containing LPCs were also directly related to markers of inflammation at week 48. We observed a direct relationship between the mol% of LPC 14:0 and TNFR1 (r = 0.33, *P* = 0.05) and sCD14 (r= 0.43, *P* = 0.01); the mol% of LPC 20:0 was directly related to levels of sCD14 (r = 0.38, *P* = 0.03), D-dimer (r = 0.46, *P* = 0.005), and TNFR1 levels (r = 0.4, *P* = 0.02). The proportional representation of LPC molecules that contain palmitic acid (16:0) was directly related to sTNFR1 (r = 0.46, *P* = 0.0058) and the percentage of CD4+Ki67+ cells (r = 0.39, *P* = 0.02, Figure 4C); levels of stearic acid (18:0) containing LPC were related to TNFR1 levels (r = 0.35, *P* = 0.03, Figure 4C).

The proportional representations of many PUFA-containing LPCs were inversely related to immune activation markers. The proportions of LPC 20:5, 22:5, and 22:6, were directly related to CD4+ T-cell counts (*P* < 0.01 for all). The proportion of LPC molecules containing 18:2 was inversely related to TNFR1 (r = -0.42, *P* = 0.0125, Figure 4C). The proportional representation of LPC 18:3 was inversely related to levels of D-Dimer and IL-6, the proportions of CD38+HLA-DR+ CD4+ (Figure 4C) and CD8+ cells, and the proportion of Ki67+ CD4+ T cells (not shown). In general, LPC molecules containing SaFAs were directly associated with markers of immune activation, while PUFA-containing LPC molecules were inversely related to levels of immune activation. Representative data for associations among LPC molecules and levels of TNFR1 are shown in Figure 4D.

## DISCUSSION

This is a substudy of A5248, in which we previously reported longitudinal reductions in viremia [27] and immune activation [28, 30], and improvement in lipid particle size and cholesterol efflux [29] among study participants initiating ART; many of these indices improved over time, but did not approach levels measured in HIV- populations. Here, we extend those findings to report changes in SaFA, MUFA, and PUFA concentrations (μM) and composition (mol%) among total, free (non-esterified), and LPC lipid molecules, and associations among these indices and immune activation.

Infection with HIV and treatment with ART can alter lipid profiles [1, 2], but earlier studies have provided only a crude assessment of changes in lipids. Here, we measured the concentrations of lipid species and fatty acid composition of plasma samples in HIV+ individuals at baseline and 48 weeks following ART and compared these levels to those of an HIV-uninfected population. We have demonstrated that total and non-esterified SaFA levels and LPC molecules enriched for SaFAs may contribute to a pro-inflammatory environment in HIV infection, even during suppressive ART, and this may contribute to the development and progression of comorbidities associated with this population. The relationship between lipids and inflammation is complex; lipids, especially SaFAs [10] and oxidized lipids [22, 23], can promote inflammation, and chronic inflammation can alter lipid processing and transport [20].

The relationship between lipids and inflammation is complicated further by MUFAs and PUFAs, as these unsaturated lipids may reduce inflammation [13–15, 17]. Our findings corroborate that PUFAs within total fatty acids, free fatty acids, and LPC molecules appear to be anti-inflamma-tory, based on several inverse relationships among levels of PUFAs and inflammatory markers, as well as our finding that greater increases in PUFA levels were associated with greater declines in sCD14 levels. We report that the concentrations and the proportional representation of many SaFAs are increased, and the PUFAs are decreased, in ART-naive participants compared to those indices among HIV- participants. Following 48 weeks of ART, levels of many PUFAs, including EPA (20:5), DPA (22:5), and DHA (22:6), increased significantly and often approached levels measured in HIV- participants.

Levels of SaFAs, MUFAs, and PUFAs may be involved in the development and progression of several disease states, as levels of these lipids are altered among individuals with CVD, diabetes, metabolic syndrome, non-alcoholic steatohepatitis (NASH), and obesity in HIV- populations [4–8]. Furthermore, levels of stearic acid and total SaFAs are increased in patients with diabetes, while levels of total PUFAs are lower in these patients than among controls [7]. Serum levels of ω3 PUFAs, including EPA, are inversely related to soft plaque scores [5], and increased concentrations of PUFAs are associated with a decreased risk of non-fatal myocardial infarction in women [8] and an overall decrease in CVD risk, based on a meta-analysis by the American Heart Association [9].

Previous studies have also reported mechanistic details about potential pro- and anti-inflammatory effects of SaFAs and PUFAs, providing insights into how these lipids may directly alter disease courses. Exposure of myeloid cells in vitro to SaFAs, specifically stearic acid, can activate the inflammasome and induce IL-1β release [10]. Palmitic acid can also activate a macrophage cell line to express TNF-α by a TLR4 and NFKB dependent mechanism [11, 35]. Polyunsaturated fatty acids, on the other hand, including EPA and DHA, can inhibit inflammasome activation and induction of TNFα and IL-1β by TLR ligands [14]. Chronic inflammation underlies the progression of many diseases, including CVD and HIV [36]; and an imbalance in SaFAs and PUFAs may contribute to chronic inflammation [10]. Modulation of innate immune signaling by PUFAs may be important in HIV infection, as microbial translocation and recognition of microbial products by TLRs has been implicated in the persistence of inflammation during virologic control [36]. The temporal and causal relationships among changes in the lipidome and changes in inflammatory biomarkers cannot be adequately assessed in this preliminary study, but should be explored in the future. Of interest, more well-defined positive associations among SaFAs and inflammation, and inverse associations among PUFAs and inflammation, were identified at week 48 than were seen at baseline. This is potentially due to ART-induced reductions in other drivers of immune activation (ie, HIV replication, lymphocytopenia, and LPS) [28].

We also measured levels of LPC, a lipid class that contains one variable fatty acid chain and is derived from the partial hydrolysis of phosphatidylcholine. Levels of LPC are increased in several diseases, including CVD [16, 17] and diabetes [19]. We report for the first time, that the concentration of LPC was elevated in treatment naive, HIV+ participants and remains high following 48 weeks of ART compared to levels in HIV- participants. The levels in HIV+ participants are similar to those reported in HIV- patients with diabetes (~200 μM) [19]. The fatty acid composition of LPC molecules likely plays a role in function; LPC molecules that contain SaFAs are often pro-inflammatory, while LPC containing PUFAs can be anti-inflammatory [17]. The fatty acid composition of LPC has also been associated with CVD; species of LPC that contain PUFAs (20:3, 20:4, 22:6) were positively associated with stable, versus unstable, coronary artery disease [37]. Here, we demonstrate that among HIV+ participants, LPC molecules are enriched for SaFAs, including LPC 16:0, 17:0, and 18:0, while levels of PUFA containing LPC molecules are decreased, compared to levels in HIV- participants. Treatment with ART tended to decrease levels of SaFA containing LPCs and increase LPC containing PUFAs; however, levels of these individual LPC molecules did not often reach levels measured in HIV- participants.

Lysophosphatidylcholine may play several important roles in modulating immune activation [17]; LPC is a component of oxLDL [21], a molecule that induces inflammatory cytokine expression through activation of the inflammasome [22, 23]. Vascular smooth muscle cells [38] and endothelial cells [34] can be activated by LPC to express pro-inflammatory cytokines. Human monocyte-derived dendritic cells can also be activated by LPC containing palmitic (16:0) and stearic acid (18:0) to increase costimulatory molecule expression and production of IL-8, IP-10, and MIP-1β [33]. In mice, intraperitoneal injection of saturated fatty acid (16:0 and 18:0) containing LPC molecules resulted in increased peritoneal inflammatory cytokine levels and leukocyte migration into tissue sites; administration of PUFA-enriched LPC (20:4, and 22:6) blocked these effects [33].

In our current study, we report direct relationships among the concentrations and composition of several SaFAs among “free”, “total”, and LPC molecules and markers of immune activation and inflammation (including IL-6, sCD14, D-dimer, TNFR-1) that are predictive of morbidity and mortality in HIV infection [39–42]. In addition, levels of PUFAs were inversely related to markers of immune activation, and following 48 weeks of ART, greater increases in DPA (22:5) and DHA (22:6) were associated with greater decreases in sCD14 levels. While these associations do not prove causality, SaFAs and PUFAs have been reported to modulate activation of monocyte and endothelial cells [10, 12, 15], cell types that may be crucial for the development and progression of CVD. Thus, additional study is warranted to determine whether increases in PUFA levels directly modulate innate immune signaling in monocytes, reducing cellular activation. We have reported previously that levels of inflammation and immune activation improved following 48 weeks of ART, but did not often reach levels measured in HIV- participants, despite undetectable viral loads [28, 30]. Additional study is also needed to determine the extent to which alterations in the lipid species we report here account for residual inflammation in ART-treated populations. As we and others have shown, increased monocyte and endothelial cell activation in HIV+ individuals [43–47], detailed characterization of the *in vivo* and *in vitro* relationships among fatty acid species, and cellular activation may help elucidate the determinants of the increased risk of CVD reported in HIV infection. In our own preliminary, unpublished, *in vitro* work, we find that palmitic and stearic acid tend to enhance LPS-induced activation of endothelial cells and myeloid cells, while LPC molecules enriched for PUFAs tend to inhibit the effects of LPS.

This study has some limitations. First, we do not have information about socioeconomic status or the dietary intake of our study participants; diet likely plays an important role in plasma lipid profiles, but as we are comparing lipid levels in the same HIV+ participants over time, dramatic changes in diet are not likely. Similarly, we do not have information on body composition or metabolic hormone status for our participants, 2 aspects of cardiometabolic health that are affected by HIV infection and ART [48]. Our HIV- and HIV+ groups are not ideally matched, but we are encouraged by the findings that ART improved the lipid profile of the baseline HIV+ participants, causing week-48 profiles to be more similar to those measured in our HIV- participants. We predicted this outcome; that ART improves, but does not often “normalize” lipid profiles in individuals with persistent low level inflammation. The parent study was not powered to explore relationships among these lipid species and immune activation, and we did not correct for multiple comparisons in this initial, exploratory investigation. In addition, we do not have information on whether the participants in both groups were concurrently taking other immunomodulatory medications such as statins and aspirin. Because the HIV+ participants were all initiating the same ART regimen, we also do not know if similar findings would be seen in ART-treated individuals or whether or not other ART regimens would induce similar changes in lipid levels. Thus, findings of modest nominal statistical significance should be viewed as preliminary and exploratory, and drawing causal linkages between changes in lipids and inflammation should be done cautiously. Nonetheless, our findings suggest SaFAs in plasma, and particularly within the LPC class, may contribute to inflammation/immune activation in HIV infection, and that increases in PUFAs may contribute to decreasing inflammation following ART. Further studies investigating the complex interactions among lipid species, inflammation, and morbid outcomes in HIV infection are warranted.

## Figures and Tables

**Supplemental Table 1A:** **Supplemental Table 1. The concentration of total and free fatty acids is altered in HIV infection at baseline and week 48 compared to levels in HIV-uninfected donors.** Plasma samples were thawed and the concentration (μM) of A) total and B) free (non-esterified) fatty acids were measured by the Complex Lipid Panel (Metabolon). Several changes in the concentrations of SaFAs, MUFAs, and PUFAs were measured among samples from HIV-uninfected participants, and in samples from HIV-infected individuals pre-ART initiation (baseline) and following 48 weeks of ART. Mean levels and standard deviations are reported. Statistically significant differences among the participant groups are designated in bold text; an exploratory value of *P* < 0.05 is used for a cutoff of significance.

Type	Lipid Number	Common Name	HIV- (n = 13		HIV+ Baseline (n = 35)		HIV+ Week 48 (n = 35)		*P* values		
			Concentraton (μM)	StD	Concentraton (μM)	StD	Concentraton (μM)	StD	HIV- v HIV+ Baseline	HIV- v HIV+ Week 48	HIV + Baseline v Week 48
SaFA	Total[FA12:0]	Lauric Acid	16.12	7.46	33.96	33.54	39.77	55.93	**0.0051**	**0.019**	0.58
SaFA	Total[FA14:0]	Myristc Acid	170.22	84.34	198.50	123.33	215.48	175.26	0.37	0.24	0.46
SaFA	Total[FA15:0]	Pentadecylic Acid	36.04	14.00	41.64	23.95	41.30	29.86	0.32	0.33	0.86
SaFA	Total[FA16:0]	Palmitc Acid	2663.13	573.89	2654.08	924.25	2795.67	1153.88	0.97	0.60	0.31
SaFA	Total[FA17:0]	Margaric Acid	46.64	13.53	55.65	28.39	54.31	29.26	0.15	0.22	0.73
SaFA	Total[FA18:0]	Stearic Acid	722.38	163.34	714.94	218.48	711.97	241.36	0.90	0.87	0.93
SaFA	Total[FA20:0]	Arachidic Acid	65.11	92.69	34.50	11.57	36.40	12.10	0.26	0.29	0.24
SaFA	Total[FA22:0]	Behenic Acid	52.26	15.15	39.25	11.37	44.15	10.38	**0.012**	0.093	**0.0074**
SaFA	Total[FA24:0]	Lignoceric Acid	42.78	14.65	30.75	8.51	33.58	7.39	**0.014**	**0.048**	**0.024**
SaFA	Total[FA26:0]	Cerotc Acid	0.53	0.14	0.47	1.36	0.46	0.12	0.15	0.092	0.70
											
MUFA	Total[FA14:1]	Myristoleic Acid	11.75	5.62	15.48	11.81	15.87	13.73	0.15	0.15	0.85
MUFA	Total[FA16:1]	Palmitoleic Acid	283.65	102.04	313.76	197.72	320.79	205.12	0.50	0.41	0.81
MUFA	Total[FA18:1]	Oleic Acid	2357.10	502.91	2702.77	1136.79	2579.16	1121.12	0.15	0.35	0.39
MUFA	Total[FA20:1]	Gondoic Acid	26.10	10.59	25.30	12.57	22.96	7.73	0.83	0.34	0.096
MUFA	Total[FA22:1]	Erucic Acid	35.47	6.29	25.90	6.70	28.84	6.79	**0.00013**	**0.0042**	**0.0029**
MUFA	Total[FA24:1]	Nervonic Acid	75.46	12.42	61.58	17.97	64.74	15.70	**0.0050**	**0.020**	0.14
MUFA	Total[FA26:1]	Hexacosanoic Acid	0.95	0.21	0.75	0.22	0.78	0.22	**0.0089**	**0.025**	0.36
											
PUFA	Total[FA18:2]	Linoleic Acid	3763.40	871.88	3341.16	909.24	3390.61	834.08	0.15	0.20	0.67
PUFA	Total[FA18:3]	α-Linolenic Acid	163.59	38.41	169.58	2.99	174.45	94.37	0.75	0.57	0.74
PUFA	Total[FA18:4]	Stearidonic Acid	2.38	1.21	1.74	1.14	2.01	0.96	0.11	0.34	0.28
PUFA	Total[FA20:2]	Eicosadienoic Acid	20.73	4.25	24.78	10.61	23.61	8.70	**0.065**	0.13	0.38
PUFA	Total[FA20:3]	Dihomo-γ-linolenic Acid	138.54	33.48	142.23	55.31	148.12	60.14	0.78	0.49	0.52
PUFA	Total[FA20:4]	Arachidonic Acid	695.40	144.85	548.13	194.01	615.45	239.93	**0.0082**	0.17	**0.015**
PUFA	Total[FA20:5]	Eicosapentanoic Acid	60.60	16.62	44.03	28.05	61.12	35.16	**0.017**	0.95	**0.0056**
PUFA	Total[FA22:2]	Docosadienoic Acid	2.64	5.33	1.18	0.60	1.20	0.31	0.34	0.35	0.82
PUFA	Total[FA22:4]	Docosatetraenoic Acid	18.54	6.13	20.16	7.59	20.43	9.92	0.45	0.43	0.82
PUFA	Total[FA22:5]	Docosapentaenoic Acid	51.67	11.14	51.35	17.08	57.81	22.01	0.94	0.21	**0.031**
PUFA	Total[FA22:6]	Docosahexaenoic Acid	144.96	52.61	115.92	56.99	141.66	72.49	0.11	0.86	**0.0045**

**Supplemental Table 1B:** 

Type	Lipid Number	Common Name	HIV- (n = 13)		HIV+ Baseline (n = 35)		HIV+ Week 48 (n = 35)		*P* values		
			Concentraton (μM)	StD	Concentraton (μM)	StD	Concentraton (μM	StD	HIV- v HIV+ Baseline	HIV- v HIV+ Week 48	HIV + Baseline v Week 48
SaFA	FFA[FA12:0]	Lauric Acid	2.24	0.44	2.13	0.87	2.49	0.99	0.59	0.23	0.14
SaFA	FFA[FA14:0]	Myristc Acid	8.39	2.49	6.79	3.49	7.96	2.42	0.088	0.60	0.13
SaFA	FFA[FA15:0]	Pentadecylic Acid	3.18	0.57	3.01	0.81	3.20	0.54	0.40	0.91	0.15
SaFA	FFA[FA16:0]	Palmitc Acid	97.48	26.35	76.75	30.87	91.26	27.01	**0.030**	0.48	**0.035**
SaFA	FFA[FA17:0]	Margaric Acid	4.70	1.00	4.49	1.02	4.86	0.97	0.52	0.62	0.095
SaFA	FFA[FA18:0]	Stearic Acid	35.98	10.62	32.15	11.39	36.75	10.25	0.29	0.82	0.083
SaFA	FFA[FA20:0]	Arachidic Acid	1.19	0.21	1.44	0.31	1.54	0.30	**0.0032**	**1.07E-04**	0.095
SaFA	FFA[FA22:0]	Behenic Acid	1.23	0.33	1.43	0.63	1.66	1.12	0.15	**0.045**	0.32
SaFA	FFA[FA24:0]	Lignoceric Acid	0.52	0.12	0.58	0.13	0.57	0.16	0.20	0.33	0.74
											
MUFA	FFA[FA14:1]	Myristoleic Acid	1.65	0.51	1.34	0.68	1.48	0.35	0.094	0.27	0.31
MUFA	FFA[FA16:1]	Palmitoleic Acid	14.13	5.67	9.62	6.47	10.85	3.95	**0.027**	0.072	0.34
MUFA	FFA[FA18:1]	Oleic Acid	127.95	50.51	87.53	50.64	105.19	41.91	**0.022**	0.16	0.11
MUFA	FFA[FA20:1]	Gondoic Acid	1.41	0.49	1.17	0.47	1.31	0.39	0.14	0.51	0.19
MUFA	FFA[FA22:1]	Erucic Acid	1.25	0.46	1.31	0.63	1.52	0.56	0.73	0.10	0.17
MUFA	FFA[FA24:1]	Nervonic Acid	0.75	0.15	0.89	0.37	0.90	0.23	0.072	**0.011**	0.85
											
PUFA	FFA[FA18:2]	Linoleic Acid	72.14	28.99	51.24	28.46	61.17	25.13	**0.037**	0.24	0.092
PUFA	FFA[FA18:3]	α-Linolenic Acid	7.03	2.85	4.80	3.18	5.81	2.30	**0.029**	0.18	0.094
PUFA	FFA[FA18:4]	Stearidonic Acid	0.31	0.06	0.28	0.06	0.30	0.04	0.100	0.61	**0.022**
PUFA	FFA[FA20:2]	Eicosadienoic Acid	1.02	0.31	0.80	0.31	0.91	0.25	**0.039**	0.24	0.097
PUFA	FFA[FA20:3]	Dihomo-γ-linolenic Acid	1.59	0.42	1.62	0.89	1.61	0.35	0.85	0.89	0.89
PUFA	FFA[FA20:4]	Arachidonic Acid	4.84	1.41	4.66	1.43	5.07	1.59	0.70	0.64	0.14
PUFA	FFA[FA20:5]	Eicosapentanoic Acid	0.94	0.18	0.92	0.19	1.06	0.24	0.83	0.056	**0.0010**
PUFA	FFA[FA22:2]	Docosadienoic Acid	0.38	0.10	0.39	0.16	0.41	0.09	0.86	0.51	0.65
PUFA	FFA[FA22:4]	Docosatetraenoic Acid	0.80	0.25	0.63	0.15	0.70	0.17	**0.042**	0.23	**0.040**
PUFA	FFA[FA22:5]	Docosapentaenoic Acid	1.51	0.44	1.24	0.36	1.40	0.35	0.060	0.41	**0.0132**
PUFA	FFA[FA22:6]	Docosahexaenoic Acid	2.21	0.80	1.56	0.74	1.97	1.00	**0.020**	0.40	**7.17E-04**

**Supplemental Table 2A:** **Supplemental Table 2. The fatty acid composition of total fatty acids and free fatty acids is altered in HIV infection at baseline and week 48 compared to levels in HIV-uninfected donors.** Plasma samples were thawed and the proportional representation (mol%) of A) total and B) free fatty acids were measured by the Complex Lipid Panel (Metabolon). Several changes in the proportional representation of SaFAs, MUFAs, and PUFAs were measured among samples from HIV- participants, and in samples from HIV+participants pre-ART initiation (baseline) and following 48 weeks of ART. Mean levels and standard deviations are reported. Statistically significant differences among the participant groups are designated in bold text; an exploratory value of *P* < 0.05 is used for a cutoff of significance.

Type	Lipid Number	Common Name	HIV- (n = 13)		HIV+ Baseline (n = 35)		HIV+ Week 48 (n = 35)		*P* values		
			Compositon (mol%)	StD	Compositon (mol%))	StD	Compositon (mol%)	StD	HIV- v HIV+ Baseline	HIV- v HIV+ Week 48	HIV + Baseline v Week 48
SaFA	Total[FA12:0]]	Lauric Acid	0.14	0.049	0.29	0.22	0.30	0.22	**0.00042**	**0.0037**	0.93
SaFA	Total[FA14:0]	Myristc Acid	1.43	0.57	1.66	0.64	1.68	0.64	0.25	0.24	0.87
SaFA	Total[FA15:0]	Pentadecylic Acid	0.31	0.11	0.36	0.13	0.34	0.13	0.22	0.35	0.56
SaFA	Total[FA16:0]	Palmitc Acid	22.78	1.75	23.18	1.50	23.75	1.50	0.47	0.12	**0.0410**
SaFA	Total[FA17:0]	Margaric Acid	0.40	0.10	0.48	0.12	0.45	0.12	**0.035**	0.12	0.18
SaFA	Total[FA18:0]	Stearic Acid	6.18	0.81	6.33	0.69	6.15	0.69	0.57	0.88	**0.067**
SaFA	Total[FA20:0]	Arachidic Acid	0.56	0.77	0.31	0.050	0.32	0.050	0.26	0.28	0.27
SaFA	Total[FA22:0]	Behenic Acid	0.45	0.11	0.36	0.092	0.40	0.092	**0.014**	0.13	**0.012**
SaFA	Total[FA24:0]	Lignoceric Acid	0.37	0.11	0.28	0.070	0.31	0.070	**0.018**	0.071	**0.040**
SaFA	Total[FA26:0]	Cerotc Acid	0.0050	0.0010	0.0040	0.0010	0.0040	0.0010	0.37	0.23	0.64
											
MUFA	Total[FA14:1]	Myristoleic Acid	0.10	0.034	0.13	0.055	0.12	0.06	**0.045**	**0.065**	0.87
MUFA	Total[FA16:1]	Palmitoleic Acid	2.41	0.69	2.60	0.89	2.62	0.89	0.44	0.37	0.88
MUFA	Total[FA18:1]	Oleic Acid	20.17	1.59	23.15	2.83	21.72	2.83	**4.77E-05**	**0.015**	**0.0093**
MUFA	Total[FA20:1]	Gondoic Acid	0.23	0.086	0.22	0.045	0.20	0.045	0.75	0.29	**0.013**
MUFA	Total[FA22:1]	Erucic Acid	0.31	0.054	0.24	0.061	0.26	0.061	**7.18E-04**	**0.011**	**0.015**
MUFA	Total[FA22:1]	Nervonic Acid	0.66	0.11	0.57	0.16	0.59	0.16	**0.031**	0.11	0.30
MUFA	Total[FA26:1]	Hexacosanoic Acid	0.0080	0.0030	0.0070	0.0020	0.0070	0.0020	0.096	0.15	0.53
											
PUFA	Total[FA18:2]	Linoleic Acid	32.21	3.97	29.93	3.82	30.08	4.42	0.090	0.12	0.83
PUFA	Total[FA18:3]	α-Linolenic Acid	1.41	0.28	1.43	0.39	1.44	0.36	0.85	0.76	0.87
PUFA	Total[FA18:4]	Stearidonic Acid	0.021	0.0090	0.015	0.0060	0.017	0.0060	0.075	0.29	0.16
PUFA	Total[FA20:2]	Eicosadienoic Acid	0.18	0.031	0.21	0.047	0.21	0.037	**0.0055**	**0.025**	0.16
PUFA	Total[FA20:3]	Dihomo-γ-linolenic Acid	1.19	0.22	1.26	0.30	1.28	0.32	0.43	0.30	0.69
PUFA	Total[FA20:4]	Arachidonic Acid	6.07	1.35	4.93	1.30	5.34	1.47	**0.016**	0.12	**0.041**
PUFA	Total[FA20:5]	Eicosapentanoic Acid	0.54	0.17	0.39	0.22	0.52	0.24	**0.017**	0.72	**0.0035**
PUFA	Total[FA22:2]	Docosadienoic Acid	0.02	0.04	0.01	0.0040	0.011	0.0030	0.35	0.36	0.76
PUFA	Total[FA22:4]	Docosatetraenoic Acid	0.16	0.047	0.18	0.042	0.18	0.059	0.24	0.36	0.73
PUFA	Total[FA22:5]	Docosapentaenoic Acid	0.45	0.12	0.46	0.085	0.50	0.093	0.94	0.27	**0.027**
PUFA	Total[FA22:6]	Docosahexaenoic Acid	1.26	0.47	1.05	0.50	1.24	0.56	0.18	0.87	**0.0016**

**Supplemental Table 2B.** Composition of Free Fatty Acids.

Type	Lipid Number	Common Name	HIV- (n = 13)		HIV+ Baseline (n = 35)		HIV+ Week 48 (n = 35)		*P* values		
			Compositon (mol%)	StD	Compositon (mol%)	StD	Compositon (mol%)	StD	HIV- v HIV+ Baseline	HIV- v HIV+ Week 48	HIV + Baseline v Week 48
SaFA	FFA[FA12:0]	Lauric Acid	0.61	0.16	0.78	0.30	0.75	0.29	**0.017**	**0.042**	0.69
SaFA	FFA[FA12:0]	Myristc Acid	2.19	0.35	2.32	0.41	2.35	0.55	0.29	0.26	0.77
SaFA	FFA[FA15:0]	Pentadecylic Acid	0.87	0.23	1.12	0.44	0.97	0.23	**0.014**	0.21	**0.042**
SaFA	FFA[FA16:0]	Palmitc Acid	25.28	2.26	26.20	1.80	26.17	2.20	0.21	0.24	0.95
SaFA	FFA[FA17:0]	Margaric Acid	1.27	0.32	1.64	0.39	1.45	0.31	**0.0025**	0.092	**0.016**
SaFA	FFA[FA18:0]	Stearic Acid	9.28	1.04	11.17	1.46	10.64	1.37	**2.25E-05**	**9.50E-04**	0.061
SaFA	FFA[FA20:0]	Arachidic Acid	0.32	0.08	0.54	0.17	0.46	0.11	**5.71E-07**	**5.27E-05**	**0.012**0.81
SaFA	FFA[FA22:0]	Behenic Acid	0.32	0.06	0.52	0.25	0.51	0.39	**6.88E-05**	**0.0096**	0.81
SaFA	FFA[FA24:0]	Lignoceric Acid	0.14	0.04	0.22	0.09	0.18	0.07	**4.27E-05**	**0.046**	**0.013**
											
MUFA	FFA[FA14:1]	Myristoleic Acid	0.43	0.09	0.46	0.11	0.44	0.13	0.30	0.73	0.36
MUFA	FFA[FA16:1]	Palmitoleic Acid	3.59	0.79	3.10	0.60	3.07	0.52	0.061	**0.045**	0.82
MUFA	FFA[FA18:1]	Oleic Acid	31.62	3.11	28.15	3.30	29.20	3.12	**0.0026**	**0.026**	0.15
MUFA	FFA[FA20:1]	Gondoic Acid	0.36	0.04	0.41	0.06	0.38	0.05	**0.0025**	0.15	**0.011**
MUFA	FFA[FA22:1]	Erucic Acid	0.32	0.07	0.45	0.15	0.43	0.09	**2.35E-04**	**8.23E-05**	0.52
MUFA	FFA[FA24:1]	Nervonic Acid	0.20	0.04	0.33	0.19	0.27	0.05	**2.88E-04**	**6.27E-05**	**0.034**
											
PUFA	FFA[FA18:2]	Linoleic Acid	17.89	2.02	16.63	2.37	17.11	2.41	0.079	0.27	0.27
PUFA	FFA[FA18:3]	α-Linolenic Acid	1.75	0.21	1.55	0.38	1.64	0.33	**0.029**	0.21	0.15
PUFA	FFA[FA18:4]	Stearidonic Acid	0.08	0.02	0.10	0.03	0.09	0.03	**0.024**	0.31	0.072
PUFA	FFA[FA20:2]	Eicosadienoic Acid	0.26	0.02	0.28	0.04	0.26	0.04	0.097	0.93	0.068
PUFA	FFA[FA20:3]	Dihomo-γ-linolenic Acid	0.42	0.07	0.62	0.45	0.48	0.12	**0.015**	**0.028**	**0.049**
PUFA	FFA[FA20:4]	Arachidonic Acid	1.26	0.22	1.70	0.54	1.51	0.54	**2.29E-04**	**0.028**	0.085
PUFA	FFA[FA20:5]	Eicosapentanoic Acid	0.25	0.06	0.35	0.13	0.33	0.10	**0.0012**	**0.0060**	0.36
PUFA	FFA[FA22:2]	Docosadienoic Acid	0.10	0.02	0.14	0.03	0.12	0.02	**4.12E-05**	**0.012**	**0.0020**
PUFA	FFA[FA22:4]	Docosatetraenoic Acid	0.21	0.04	0.23	0.06	0.21	0.04	0.137	0.98	**0.030**
PUFA	FFA[FA22:5]	Docosapentaenoic Acid	0.40	0.08	0.45	0.14	0.42	0.10	0.095	0.51	0.11
PUFA	FFA[FA22:6]	Docosahexaenoic Acid	0.58	0.17	0.54	0.16	0.58	0.22	0.53	0.98	0.21

**Supplemental Table 3A:** **Supplemental Table 3. The fatty acid composition and concentration of lysophosphatidylcholine (LPC) molecules is altered in HIV infection at baseline and week 48 compared to levels in HIV-uninfected donors.** Plasma samples were thawed and A) the proportional representation (mol%) and B) concentration (μM) of LPC molecules were measured by the Complex Lipid Panel (Metabolon). Several changes in the proportional representation of SaFAs, MUFAs, and PUFAs were measured among samples from HIV- participants, and in samples from HIV+ participants pre-ART initiation (baseline) and following 48 weeks of ART. Mean levels and standard deviations are reported. Statistically significant differences among the participant groups are designated in bold text; an exploratory value of *P* < 0.05 is used for a cutoff of significance.

Type	Lipid Number	Common Name	HIV- (n = 13)		HIV+ Baseline (n = 35)		HIV+ Week 48 (n = 35)		*P* values		
			Concentraton (μM)	StD	Concentraton (μM)	StD	Concentraton (μM)	StD	HIV- v HIV+ Baseline	HIV- v HIV+ Week 48	HIV + Baseline v Week 48
SaFA	LPC[FA12:0]	Lauric Acid	0.010	0.012	0.0040	0.0020	0.0090	0.0040	0.39	0.87	0.089
SaFA	LPC[FA14:0]	Myristc Acid	0.12	0.060	0.15	0.11	0.19	0.089	0.18	**0.0040**	**0.033**
SaFA	LPC[FA15:0]	Pentadecylic Acid	0.49	0.17	0.65	0.33	0.70	0.31	**0.035**	**0.0050**	0.31
SaFA	LPC[FA16:0]	Palmitc Acid	87.90	25.46	111.83	40.27	120.95	36.46	**0.020**	**0.0013**	0.092
SaFA	LPC[FA17:0]	Margaric Acid	1.36	0.36	1.96	0.75	2.053	0.66	**5.20E-04**	**3.46E-05**	0.40
SaFA	LPC[FA18:0]	Stearic Acid	18.70	5.31	27.94	10.88	27.55	8.78	**3.18E-04**	**1.53E-04**	0.79
SaFA	LPC[FA20:0]	Arachidic Acid	0.056	0.016	0.070	0.026	0.061	0.022	**0.034**	0.36	0.086
SaFA	LPC[FA22:0]	Behenic Acid	0.0070	0.0040	0.0060	0.0030	0.0080	0.0040	0.45	0.92	0.17
SaFA	LPC[FA24:0]	Lignoceric Acid	0.0080	0.0030	0.0050	0.0020	0.0060	0.0050	0.057	0.40	0.20
											
MUFA	LPC[FA14:1]	Myristoleic Acid	0.0070	0.0060	0.0090	0.0050	0.0060	0.0030	0.54	0.70	0.14
MUFA	LPC[FA16:1]	Palmitoleic Acid	2.24	1.18	2.50	1.051	2.73	0.97	0.49	0.19	0.13
MUFA	LPC[FA18:1]	Oleic Acid	21.81	10.41	21.98	7.54	22.66	6.60	0.96	0.79	0.54
MUFA	LPC[FA20:1]	Gondoic Acid	0.14	0.043	0.19	0.077	0.19	0.070	**0.0065**	**0.0062**	0.87
MUFA	LPC[FA22:1]	Erucic Acid	0.010	0.007	0.0080	0.0060	0.0070	0.0050	0.62	0.50	0.75
MUFA	LPC[FA24:1]	Nervonic Acid	0.012	0.011	0.0070	0.0050	0.0050	0.0020	0.39	0.25	0.91
											
PUFA	LPC[FA18:2]	Linoleic Acid	37.076	16.42	27.15	10.29	28.81	9.44	0.059	0.11	0.33
PUFA	LPC[FA18:3]	α-Linolenic Acid	0.30	0.21	0.23	0.098	0.27	0.12	0.26	0.64	0.079
PUFA	LPC[FA18:4]	Stearidonic Acid	0.0060	0.0020	0.0070	0.006	0.0070	0.0040	0.63	0.52	0.24
PUFA	LPC[FA20:2]	Eicosadienoic Acid	0.34	0.18	0.39	0.15	0.35	0.15	0.41	0.81	0.23
PUFA	LPC[FA20:3]	Dihomo-γ-linolenic Acid	2.29	1.32	2.53	1.28	2.41	0.87	0.58	0.76	0.58
PUFA	LPC[FA20:4]	Arachidonic Acid	0.40	1.39	3.73	1.58	3.95	1.45	0.51	0.83	0.39
PUFA	LPC[FA20:5]	Eicosapentanoic Acid	0.15	0.055	0.115	0.085	0.150	0.083	0.12	0.96	0.061
PUFA	LPC[FA22:2]	Docosadienoic Acid	0.0070	0.0050	0.0060	0.0050	0.0080	0.0050	0.87	0.62	0.11
PUFA	LPC[FA22:4]	Docosatetraenoic Acid	0.066	0.040	0.093	0.059	0.080	0.052	0.075	0.31	0.30
PUFA	LPC[FA22:5]	Docosapentaenoic Acid	0.192	0.085	0.215	0.113	0.225	0.095	0.45	0.26	0.64
PUFA	LPC[FA22:6]	Docosahexaenoic Acid	0.29	0.12	0.27	0.14	0.32	0.16	0.58	0.49	0.099

**Supplemental Table 3B:** 

Type	Lipid Number	Common Name	HIV- (n = 13)		HIV+ Baseline (n = 35)		HIV+ Week 48 (n = 35)		*P* values		
			Compositon (mol%)	StD	Concentraton	StD	Compositon (mol%)	StD	HIV- v HIV+ Baseline	HIV- v HIV+ Week 48	HIV + Baseline v Week 48
SaFA	LPC[FA12:0]	Lauric Acid	0.008	0.009	0.002	0.001	0.005	0.003	0.3266	0.5996	0.2919
SaFA	LPC[FA14:0]	Myristc Acid	0.067	0.029	0.072	0.029	0.087	0.025	0.6399	**0.0462**	**0.0165**
SaFA	LPC[FA15:0]	Pentadecylic Acid	0.284	0.091	0.317	0.096	0.332	0.094	0.2737	0.2116	0.7408
SaFA	LPC[FA16:0]	Palmitc Acid	50.042	4.539	55.277	4.693	56.399	4.113	**0.0019**	**0.0003**	0.0787
SaFA	LPC[FA17:0]	Margaric Acid	0.778	0.135	0.965	0.172	0.954	0.150	**0.0005**	**0.0007**	0.6723
SaFA	LPC[FA18:0]	Stearic Acid	10.724	1.964	13.785	1.626	12.873	1.804	**0.0001**	**0.0026**	**0.0004**
SaFA	LPC[FA20:0]	Arachidic Acid	0.032	0.008	0.036	0.012	0.029	0.009	0.3177	0.2367	**0.0133**
SaFA	LPC[FA22:0]	Behenic Acid	0.005	0.003	0.003	0.001	0.004	0.002	0.2204	0.4191	0.1874
SaFA	LPC[FA24:0]	Lignoceric Acid	0.004	0.002	0.002	0.001	0.003	0.002	0.0592	0.1912	0.1640
											
MUFA	LPC[FA14:1]	Myristoleic Acid	0.004	0.004	0.004	0.002	0.003	0.002	0.9659	0.3991	0.1167
MUFA	LPC[FA16:1]	Palmitoleic Acid	1.226	0.297	1.219	0.244	1.265	0.240	0.9439	0.6700	0.3306
MUFA	LPC[FA18:1]	Oleic Acid	11.990	1.881	10.885	1.296	10.640	1.463	0.0680	**0.0313**	0.2703
MUFA	LPC[FA20:1]	Gondoic Acid	0.078	0.020	0.093	0.021	0.088	0.026	**0.0407**	0.1989	0.3561
MUFA	LPC[FA22:1]	Erucic Acid	0.006	0.005	0.004	0.004	0.003	0.002	0.5120	0.2240	0.3894
MUFA	LPC[FA24:1]	Nervonic Acid	0.007	0.006	0.004	0.002	0.003	0.001	0.3029	0.1949	0.8425
											
PUFA	LPC[FA18:2]	Linoleic Acid	20.426	3.979	13.587	3.791	13.703	3.936	**0.00003**	**0.00003**	0.8332
PUFA	LPC[FA18:3]	γ-Linolenic Acid	0.160	0.057	0.114	0.042	0.128	0.046	**0.0169**	0.0815	0.1460
PUFA	LPC[FA18:4]	Stearidonic Acid	0.003	0.001	0.004	0.003	0.004	0.002	0.8744	0.7580	0.2968
PUFA	LPC[FA20:2]	Eicosadienoic Acid	0.183	0.058	0.193	0.042	0.165	0.048	0.6029	0.3250	**0.0159**
PUFA	LPC[FA20:3]	Dihomo-γ-linolenic Acid	1.254	0.337	1.236	0.470	1.129	0.300	0.8836	0.2553	0.2134
PUFA	LPC[FA20:4]	Arachidonic Acid	2.334	0.639	1.864	0.629	1.841	0.480	**0.0336**	**0.0216**	0.7866
PUFA	LPC[FA20:5]	Eicosapentanoic Acid	0.088	0.036	0.058	0.043	0.070	0.038	**0.0220**	0.1580	0.1274
PUFA	LPC[FA22:2]	Docosadienoic Acid	0.004	0.003	0.003	0.003	0.004	0.004	0.6110	0.8964	0.1057
PUFA	LPC[FA22:4]	Docosatetraenoic Acid	0.037	0.021	0.046	0.028	0.038	0.020	0.2481	0.9451	0.1250
PUFA	LPC[FA22:5]	Docosapentaenoic Acid	0.108	0.034	0.108	0.053	0.106	0.037	0.9786	0.8145	0.7999
PUFA	LPC[FA22:6]	Docosahexaenoic Acid	0.171	0.077	0.137	0.067	0.155	0.083	0.1729	0.5134	0.2314
